# Autophagy and endosomal trafficking inhibition by *Vibrio cholerae* MARTX toxin phosphatidylinositol-3-phosphate-specific phospholipase A1 activity

**DOI:** 10.1038/ncomms9745

**Published:** 2015-10-26

**Authors:** Shivani Agarwal, Hyunjin Kim, Robin B. Chan, Shivangi Agarwal, Rebecca Williamson, Wonhwa Cho, Gilbert D. Paolo, Karla J. F. Satchell

**Affiliations:** 1 Department of Microbiology-Immunology, Northwestern University, Feinberg School of Medicine, 303 E Chicago Avenue, Chicago, Illinois 60611, USA; 2 Department of Chemistry, University of Illinois at Chicago, 845 West Taylor Street, Chicago, Illinois 60607, USA; 3 Department of Pathology and Cell Biology, 630 West 168th Street, Columbia University, New York, New York 10032, USA

## Abstract

*Vibrio cholerae*, responsible for acute gastroenteritis secretes a large multifunctional-autoprocessing repeat-in-toxin (MARTX) toxin linked to evasion of host immune system, facilitating colonization of small intestine. Unlike other effector domains of the multifunctional toxin that target cytoskeleton, the function of alpha-beta hydrolase (ABH) remained elusive. This study demonstrates that ABH is an esterase/lipase with catalytic Ser–His–Asp triad. ABH binds with high affinity to phosphatidylinositol-3-phosphate (PtdIns3P) and cleaves the fatty acid in PtdIns3P at the *sn1* position *in vitro* making it the first PtdIns3P-specific phospholipase A1 (PLA1). Expression of ABH *in vivo* reduces intracellular PtdIns3P levels and its PtdIns3P-specific PLA1 activity blocks endosomal and autophagic pathways. In accordance with recent studies acknowledging the potential of extracellular pathogens to evade or exploit autophagy to prevent their clearance and facilitate survival, this is the first report highlighting the role of ABH in inhibiting autophagy and endosomal trafficking induced by extracellular *V. cholerae*.

## Abstract (web summary)

The MARTX toxin of *V. cholerae* processes itself to deliver three distinct effector domains to the cytosol. Here the authors show that the third effector domain is a phosphatidylinositol-3-phosphate (PtdIns3P)-specific phospholipase that inhibits autophagy in target cells.

## Graphical abstract

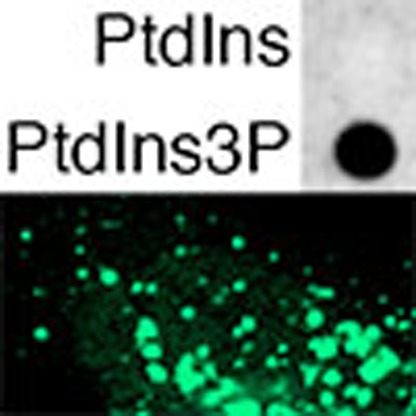

The diarrhoeal disease cholera is caused by the ingestion of food or water contaminated with the Gram-negative bacterium *Vibrio cholerae* through the action of the ADP-ribosylating cholera toxin^1^. In addition to cholera toxin, *V. cholerae* El Tor O1, the predominating strain variant responsible for cholera over the past 50 years, secretes a number of accessory toxins and proteases, including the multifunctional-autoprocessing repeats-in-toxin toxin (MARTX)^2^. This and other accessory toxins have been linked to enhanced colonization of the small intestine by facilitating evasion of host innate immune cells during early stages of bacterial infection^3^,^4^ The 4,545 amino acid (aa) MARTX toxin is secreted from the bacteria and then at least partially translocated across the eukaryotic cell plasma membrane where it delivers three effector domains by induced autoprocessing^5^,^6^,^7^. The actin cross-linking domain (ACD) causes cell rounding by introducing an isopeptide bond between protomers of G-actin^8^,^9^. The Rho inactivation domain (RID) independently induces actin cytoskeleton disassembly by inactivation of small GTPases Rho, Rac, and CDC42 (refs 10, 11, 12). The third effector domain of MARTX_Vc_, the α/β-hydrolase (ABH), has been identified as an effector domain independently released from the MARTX_Vc_ holotoxin by the cysteine protease domain (CPD)-mediated autoprocessing^5^,^7^ and by sequence homology to α/β-hydrolase family members^13^. Preliminary investigation indicate that ABH domain alters cell signalling and indirectly activates small GTPase CDC42 (ref. 12), but its effect on cell signalling is as yet unknown.

Phosphoinositides are low abundant phospholipids that serve as signals to recruit specific protein effectors to membranes resulting in activation or inactivation of cellular processes. A key phosphoinositide is phosphatidylinositol-3-phosphate (PtdIns3P), which plays a fundamental role in the endolysosomal pathway and in autophagy, where it initiates autophagosome biogenesis within cells. Autophagy is a cellular process that promotes cell survival through engulfment of intracellular aggregates and organelles for delivery to the lysosome for degradation^14^,^15^,^16^. The process is also integral to the host response to pathogens. Intracellular bacterial pathogens are known to block autophagy by a variety of mechanisms to enhance bacterial survival within a vacuole or in the cytoplasm^17^,^18^. Although long thought to be a response only to intracellular pathogens, autophagy is also recently recognized as critical to innate immune signalling during the response of cells to extracellular pathogens to promote cytokine and chemokine production and initiate bacterial clearance mechanisms^19^,^20^,^21^.

The α/β-hydrolase fold found within ABH is common to a large number of enzymes of different phylogenetic origin and catalytic functions, including esterases and lipases^22^,^23^. In this study, we show that the ABH domain of the *V. choleare* MARTX toxin is a novel phospholipase with a unique specificity for PtdIns3P, releasing free fatty acid (FFA) from the *sn*1 position. Consistent with the established role of PtdIns3P in cells, we find that this PtdIns3P-specific PLA1 activity results in loss of PtdIns3P both on endosomes and preautophagosomal structures, thus causing inhibition of autophagosome formation in response to both stress and to bacterial exposure. Thus, the MARTX toxin effector domain ABH represents a novel mechanism by which bacteria evade the host response to infection.

## Results

### The ABH domain is an esterase/lipase

To initiate study of the MARTX ABH domain, the aa sequence was aligned to the structural database. ABH was found to have 30% identity to *Coxiella burnetti* serine hydrolase (pdb 3TRD). Based on this crystal structure (Supplementary Fig. 1d), we modelled a catalytic cleft of ABH formed by Ser-3259, Asp-3338 and His-3369. Recombinant ABH (rABH) and mutant variants rABH S3259A (rABHS), D3338A (rABHD), and H3369A (rABHH) were purified. Mutant proteins showed no gross perturbations in the secondary structure in comparison to rABH, while a modest 5–7 °C decrease in *T*_m_ suggests a slight alteration in the tertiary structure, particularly for rABHD (Supplementary Fig. 1).

**Figure 1:**
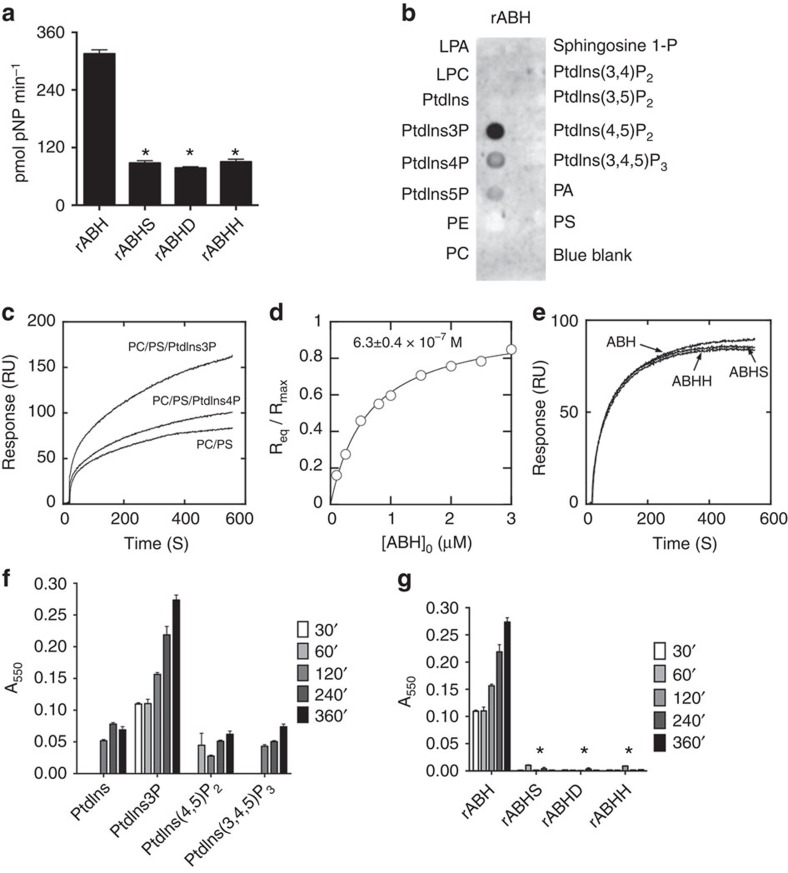
ABH binds to and cleaves PtdIns3P. (**a**) Release of pNP from the C8 substrate with the wild type and catalytic mutants of rABH in 30 min. (**b**) PIP strip precoated with indicated lipids incubated with rABH and probed with anti-His monoclonal antibody (**c**) Kinetic SPR sensorgrams of rABH binding to PC/PS (80:20), PC/PS/PtdIns3P (77:20:3), and PC/PS/PtdIns4P (77:20:3) vesicles (**d**) *K*_d_ for binding of rABH to PC/PS/PtdIns3P (77:20:3) vesicles. Solid line represents the theoretical curve with *K*_*d*_ (mean±s.d.). (**e**) Kinetic SPR sensorgrams of rABH and active site mutants, rABHS and rABHH, for PC/PS/PtdIns3P (77:20:3) vesicles. (**f**) Release of FFAs from indicated substrates with rABH and (**g**) from PtdIns3P by wild type and mutant rABH. Values represent mean±s.d. (*n*=3). For **a** and **g**, asterisk indicates statistically significant difference from rABH (*P*<0.0001).

The purified proteins were screened in an array of common assays representative of known activities of enzymes with an α/β-hydrolase fold. In the screen, rABH was found to cleave 4-nitrophenyl caprylate (pNP-C8), without requiring divalent cations, revealing ABH has esterase activity (Fig. 1a, Supplementary Fig. 2). Catalytically inactive variants of rABH were unable to hydrolyse pNP-C8, indicating ABH to be a serine esterase with a classical Ser-Asp-His catalytic triad.

To determine if ABH serine esterase has specificity for ester bonds within lipids, various lipids moieties were tested for FFA release. rABH showed a low, but detectable lipase activity on common membrane phospholipids phosphatidylcholine (PC), phosphatidylglycerol, and phosphatidyethanolamine, or diacylglycerol (DAG). A slightly increased lipase activity was observed for D-L-α-palmitin (Supplementary Fig. 2). Thus, we hypothesized that ABH might be a lipase, but with highly specific headgroup preference distinguishing it from cellular lipases and other bacterial effectors with phospholipase activity.

### rABH is a PtdIns3P-specific phospholipase

To ascertain if ABH has high specificity recognition among lipids, purified rABH proteins were overlaid overnight at 4 °C onto phospholipid-coated strips and protein binding was detected. rABH and the catalytically inactive mutants bound only to monophosphate inositol lipids, with high specificity for PtdIns3P (Fig. 1b and Supplementary Fig. 3). To quantitatively determine lipid affinity and specificity of ABH, the binding of rABH to vesicles was measured by surface plasmon resonance (SPR). rABH showed affinity for PC/phosphatidylserine (PS) (80:20) vesicles, indicating that it has affinity for anionic membranes. Addition of 3 mole-percent of PtdIns3P to the vesicles (PC/PS/PtdIns3P) (77:20:3) greatly increased rABH binding, while PtdIns4P had little effect under the same conditions (Fig. 1c). This suggests that the faint binding of rABH to PtdIns4P in the overlay assay was non-specific and establishes rABH is PtdIns3P-specific protein. Since the binding of ABH to PtdIns5P on the surface overlay blot is lesser than to PtdIns4P, it was also deemed non-specific. Equilibrium SPR analysis showed that rABH bound to PC/PS/PtdIns3P vesicles with *K*_d_=630±40 nM (Fig. 1d). Also, binding of rABHS or rABHH to PC/PS/PtdIns3P vesicles was comparable to that of wild type rABH, indicating these residues do not contribute to headgroup specificity (Fig. 1e).

As ABH binds specifically to PtdIns3P, it was assessed whether PtdIns3P is a lipase substrate of rABH. Incubation of rABH with 18:1 PtdIns3P substrate released FFA as early as 30 min and it increased significantly over time with a specific activity of 61.9±5.3 nM FFA/min/mg of enzyme. Similar release of FFA was not observed for PtdIns, PtdIns(3,4)P_2_, or PtdIns(3,4,5)P_3_ indicating that ABH is highly specific for PtdIns3P and this cleavage is dependent on the active site residues (Fig. 1f,g). This indicates that ABH is the first known phospholipase with the unique specificity of binding and cleaving only PtdIns3P.

### rABH cleaves the *sn*1 ester bond

**Figure 2:**
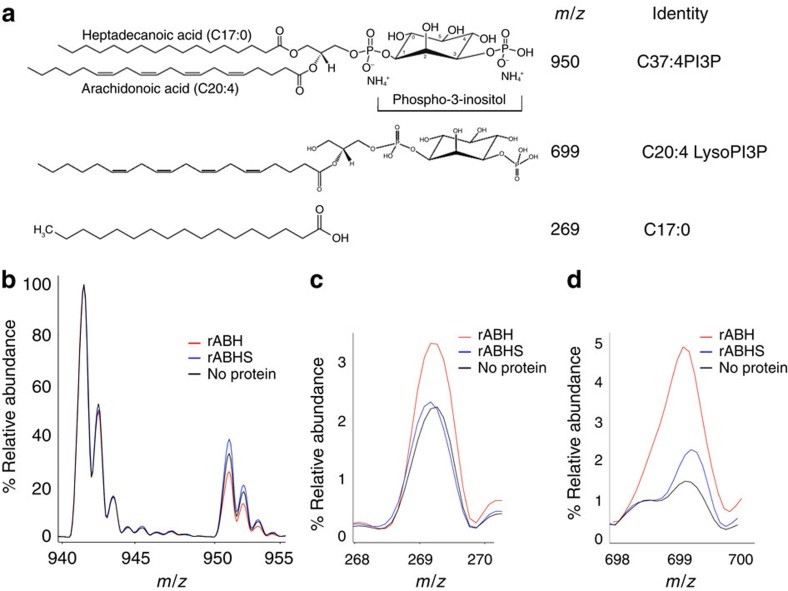
ABH is a PtdIns3P-specific PLA1. (**a**) Molecular structures of heptadecanoic acid, lyso-PtdIns3P, and PtdIns3P substrate with their respective *m/z*. (**b**–**d**) MS profile of substrate (black) or substrate treated with rABH (red) or rABHS (blue). The *m/z* for each of the peaks in each case are labelled. All spectra were normalized to the standard PtdIns3P 36:2 (941 *m/z*).

To identify whether the *sn1* or *sn2* ester bond of PtdIns3P is cleaved by ABH, the products of an *in vitro* phospholipase reaction were analysed using mass spectrometry. A C37:4 substrate comprised of PtdIns3P with distinct fatty acids heptadecanoic acid (C17:0) and arachidonic acid (C20:4) on *sn1* and *sn2* positions, respectively, was used (Fig. 2a). Superimposition of the chromatograms obtained for substrate incubated with rABH showed a significant increase in the relative abundance of a mass of 269 *m/z* (Fig. 2c), which corresponds to the reference standard for free heptadecanoic acid (C17:0; Supplementary Fig. 4). This increase in abundance required catalytically active enzyme. Coincident with the appearance of the heptadecanoic acid, there is a quantitative reduction in the relative abundance of the C37:4 substrate with a mass of 950 *m/z* (Fig. 2b) and an increase in a 699 *m/z* peak (Fig. 2d), which was confirmed by MS/MS to be lyso-PtdIns3P (C20:4) (Supplementary Fig. 4). This indicates that arachidonic acid on the *sn*2 bond remains intact with the headgroup after *sn*1 ester bond is cleaved releasing heptadecanoic acid.

Altogether, these results show that the ABH effector domain of the *V. cholerae* MARTX toxin is a PtdIns3P-specific phospholipase A1 (PLA1) that is a member of the α/β-hydrolase fold family of enzymes with a catalytic serine. This is to our knowledge the first description of a PtdIns3P-specific phospholipase A1 from any species. This further reveals that the MARTX toxin effector domain is not simply mimicking a normal mammalian cell biological activity, but using a novel mechanism of controlling PtdIns3P levels during intoxication that is not among the systems that control levels of the lipid normally.

### Inhibition of cellular autophagy by ABH

**Figure 3:**
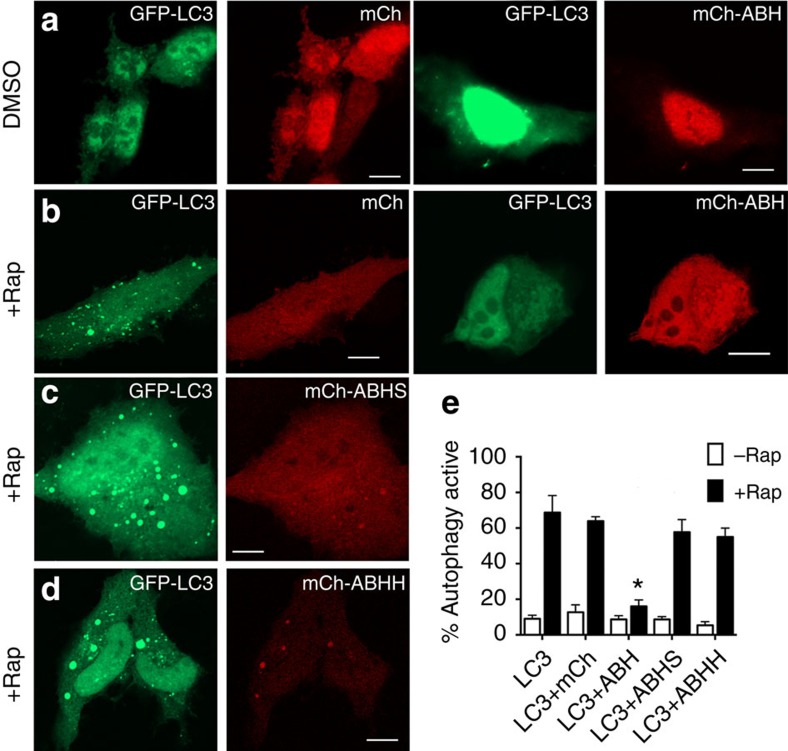
ABH inhibits rapamycin-induced autophagy. HeLa cells transfected with GFP-LC3 alone (green) or co-transfected with mCh, mCh-ABH or mutant mCh-ABHS/mCh-ABHH variants (red). The cells were treated for 12 h with either DMSO as control (**a**) or 0.4 μM rapamycin (**b**–**d**). Scale bar, 10 μm. (**e**) Autophagy-positive cells were quantified and represented as mean±s.d. (*n*=120). The cells expressing mCh-ABH showed statistically significant difference from controls cells expressing GFP-LC3, mCh, or mCh-ABHS (*P*<0.0001). Redistribution of mCh-ABH from nucleus to cytoplasm on addition of rapamycin was not consistent and varied with expression level. Redistribution of GFP-LC3 after rapamycin treatment was reproducible consistent with observations by others^57^.

To begin to understand the biological consequence of the novel PtdIns3P-specific phospholipase, the ABH domain was ectopically expressed in epithelial cells. On overexpression, ABH did not induce cell lysis or any other observable change in cell morphology (Fig. 3a). Thus, we considered the normal roles of PtdIns3P in the cell that this phospholipase might modulate, namely phagocytosis, autophagy and endosomal sorting.

Although PtdIns3P is present on phagosomes during phagocytosis^14^,^24^, our previous study showed that inhibition of phagocytosis due to ABH is only about 20%, while inhibition due to the co-present ACD of the MARTX_Vc_ toxin is 80% and accounts for all phagocytosis inhibition by the holotoxin, with and without ABH^12^. The retention of ABH in the toxin despite an overwhelming inhibition of phagocytosis by the ACD suggests that hydrolysis of PtdIns3P must be important also for another intracellular pathway.

PtdIns3P is one of the components indispensable for autophagosome biogenesis^25^,^26^. During autophagosome biogenesis, PtdIns3P is involved in spatially-restricting the lipidation of the microtubule-associated protein 1 light chain 3 (LC3) on preautophagosomal membranes through PtdIns3P-binding effectors^16^,^27^,^28^. LC3 is a mammalian homologue of Atg8 and is commonly used as a marker of autophagosomes and the differential expression and localization of LC3 on the autophagosomes is representative of autophagic activity in the cells^29^,^30^.

Consistent with this, ectopically expressed GFP-LC3 in normal HeLa cells showed uniform cytoplasmic localization. However, in cells treated with the mTORC1 pathway inhibitor rapamycin to induce autophagy, ∼80% of cells showed a redistribution of GFP-LC3 to small punctae representing autophagosomes (Supplementary Fig. 5).

Similarly, cells co-transfected to express both GFP-LC3 and mCherry (mCh) showed autophagosome formation in response to rapamycin (Fig. 3a). However, rapamycin-treated HeLa cells co-expressing GFP-LC3 with mCh fused to ABH (mCh-ABH) did not show puncta, suggesting that autophagosome formation is markedly reduced when ABH is present (Fig. 3b). In addition, autophagy induced by incubating cells in nutrient deprived Hank's Balanced Salt Solution was blocked in HeLa cells expressing mCh-ABH (Supplementary Fig. 6). In fact, these cells appeared similar to cells induced to undergo autophagy in the presence of 3-methyladenine, a known autophagy inhibitor that blocks type III phosphatidylinositol 3-kinases (PI3K; Supplementary Fig. 5).

This observed inhibition of autophagosome formation in response to rapamycin or nutrient deprivation was dependent on catalytic activity of ABH since the expression of mutant proteins mCh-ABHS or mCh-ABHH did not affect autophagosome formation in response to either stimulus (Fig. 3c–e, Supplementary Figs 6 and 7). These data suggest that the phospholipase activity of ABH inhibits autophagy induced by cell stress by hydrolysing the PtdIns3P autophagy initiator lipid.

### Catalytically inactive ABH localizes to autophagosomes

It is alternatively possible that steric hindrance due to binding of ABH to PtdIns3P simply blocks other proteins necessary for autophagosome formation from binding PtdIns3P. Since rABHS and rABHH can bind PtdIns3P *in vitro* (Fig. 1), it seemed reasonable that they would retain binding to PtdIns3P on autophagosomes. Indeed, co-localization of mutant mCh-ABH with GFP-LC3 was observed in puncta (Fig. 3c,d and Supplementary Fig. 8). As autophagosomes are clearly forming in these cells, autophagy is not inhibited in spite of the ability of the modified proteins to bind PtdIns3P. These data indicate that it is the unique phospholipase activity of ABH, not phospholipid binding, which mediates inhibition of autophagosome formation.

### ABH-mediated defects in endosomal sorting

In addition to autophagy, another key function of PtdIns3P is to control the sorting of cargo into intraluminal vesicles (ILVs) of multivesicular endosomes^14^. To test whether ABH affects endosomal sorting also, we visualized the intraluminal localization of amyloid precursor protein (APP) in cells expressing active ABH and catalytically inactive ABH using confocal microscopy. This cargo was previously shown to require PI3P for ILV sorting^31^. Expression of the constitutively active mutant of Rab5 (Rab5^Q79L^) in HeLa cells enlarges the endosomes and allows discrimination between the limiting membrane and ILVs of endosomes^32^. When APP-GFP is co-transfected, the ratio of APP fluorescence that is internal to the Rab5-positive endosome as opposed to peripheral localization can be used to identify ILV-sorting defects^31^.

**Figure 4:**
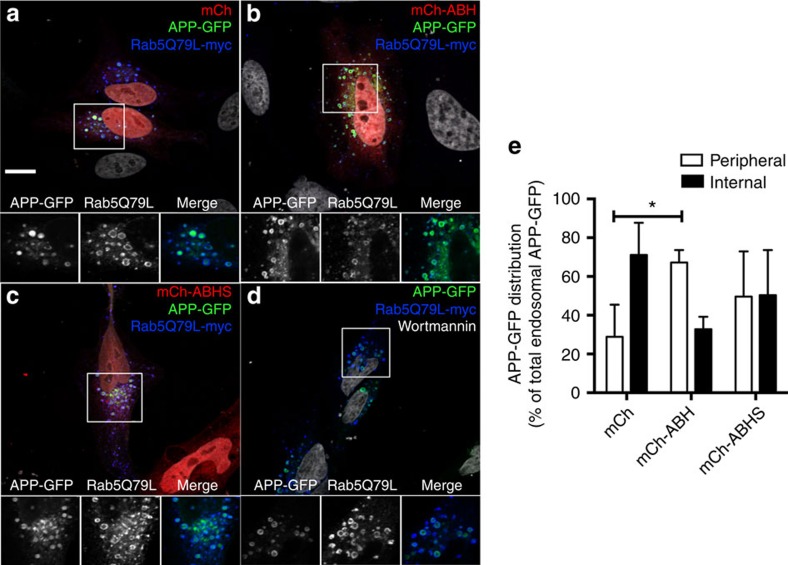
ABH blocks intraluminal sorting of APP-GFP. HeLa cells transfected with Rab5^Q79L^-myc, APP-GFP, and mCh alone (**a**) mCh-ABH (**b**) or mCh-ABHS (**c**). As a positive control, cells were treated with 100 nM wortmannin for 30 min before fixation (**d**). Cells were fixed and labelled with an anti-myc antibody (blue) and counterstained with 4,6-diamino-2-phenylindole dihydrochloride (DAPI; white) for confocal microscopy. Scale bar, 10 μm. Smaller insets show enlargement of regions indicated by box. (**e**) The localization of APP-GFP inside the endosomal lumen (internal) or on the endosome-limiting membrane (peripheral) was quantified and expressed as % of the total endosomal APP-GFP. Values denote mean±s.d. (*n*=20–24 cells from two experiments with an average quantification of seven giant endosomes per cell). *P=0.001 in peripheral localization between mCh and mCh-ABH. mCh-ABHS was not significantly different than either mCh or mCh-ABH.

As observed previously^31^, ∼70% of endosomes in Rab5^Q79L^/APP-GFP-expressing cells co-transfected to express only mCh showed luminal fluorescence within enlarged endosomes indicating association of APP-GFP with ILVs (Fig. 4a). However, only 35% of cells expressing mCh-ABH displayed luminal localization of APP-GFP, with majority of the endosomes having APP-GFP on the limiting membranes. As opposed to the wild-type ABH, the expression of ABHS showed variable results with ∼50% cells with APP-GFP being localized to the peripheral membrane of endosomes. The inhibition by mCh-ABHS was not significantly different from either mCh or mCh-ABH suggesting this has an intermediate phenotype and this more subtle effect is subject to some steric hindrance by binding of mCh-ABHS to PtdIns3P. These results show that ABH affects several PtdIns3P-dependent cellular dependent pathways including entry by phagocytosis, endosomal trafficking and autophagy.

### Intracellular PtdIns3P levels are reduced by ABH

**Figure 5:**
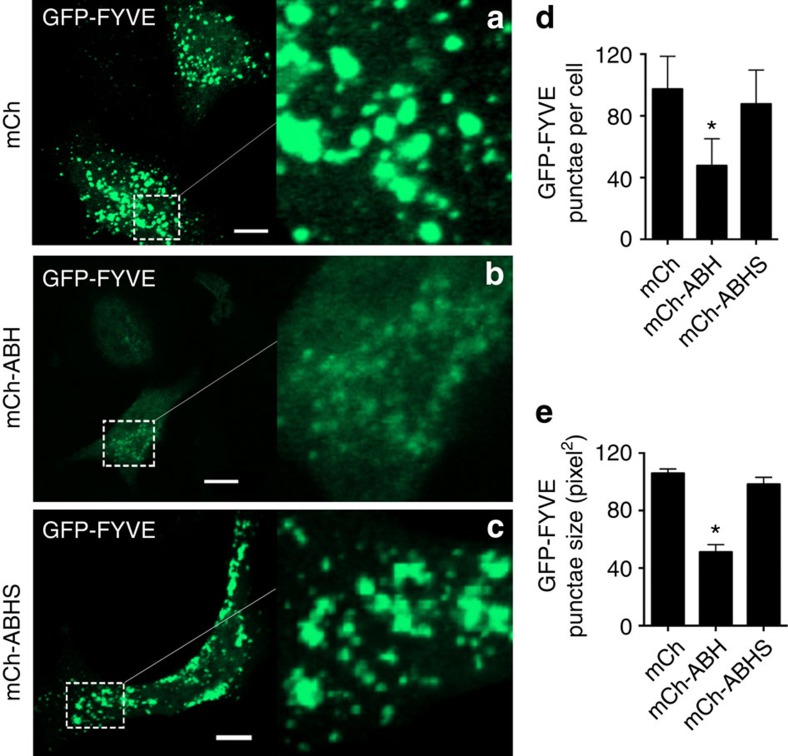
ABH affects GFP-FVYE localization to the endosomes. Rapamycin-treated HeLa cells transfected with GFP-FYVE alone (green) or co-transfected with mCh (**a**) mCh-ABH (**b**) or mCh-ABHS (**c**). Scale bar, 10 μm. Region enlarged to show puncta. (**d**,**e**) The number and size of GFP-FYVE puncta in cells represented as mean±s.d. (*n*=60). The cells expressing mCh-ABH were statistically significant from controls cells expressing GFP-FYVE, mCh or mCh-ABHS (*P*<0.0001).

In order to more directly link the *in vitro* phospholipase activity of ABH to *in vivo* defects in endocytic trafficking and autophagy, the overall cellular levels of PtdIns3P were visualized. First, the intracellular levels of PtdIns3P were quantified using PtdIns3P-binding reporter GFP-2 × FYVE (refs 33, 34; referred as GFP-FYVE). HeLa cells co-expressing GFP-FYVE and mCh-ABH, but not mCh-ABHS or mCh, showed ∼50% reduction in the size and number of GFP-FYVE punctae when autophagy was induced using rapamycin, suggesting reduced PtdIns3P throughout the cells (Fig. 5). This result indicates reduction in the cellular concentration of PtdIns3P on endosomal membranes.

**Figure 6:**
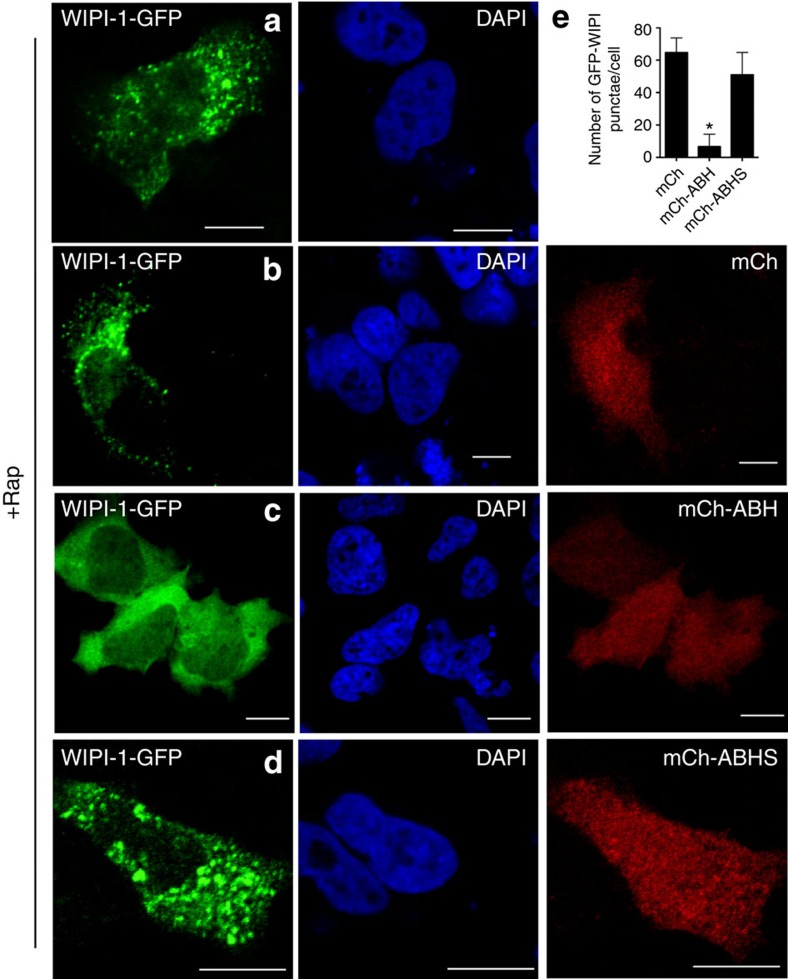
Reduced recruitment of WIPI-GFP for autophagosome biogenesis. Rapamycin-treated HeLa cells transfected with GFP-WIPI-1 alone (green) (**a**) or co-transfected with mCh (**b**) mCh-ABH (**c**) or mCh-ABHS (**d**). Scale bar, 10 μm. (**e**) The number of WIPI-1-GFP punctae in cells represented as mean±s.d. (*n*=45). The cells expressing mCh-ABH showed statistically significant difference from controls cells expressing WIPI-1-GFP, mCh or mCh-ABHS (*P*<0.005).

In a similar experiment, we assessed localization and abundance of the Atg18/WD repeat domain phosphoinositide-interacting protein 1 (WIPI-1), a PtdIns3P-binding probe but more specific for preautophagosomal structures^35^. For this PtdIns3P-binding protein, there was almost complete absence of WIPI-1-GFP punctae in mCh-ABH expressing cells, indicating a critical reduction of PtdIns3P for autophagosomes (Fig. 6).

Taken together, our results reveal that ABH can target the general PtdIns3P pool affecting endosomal sorting and phagocytosis, and it potently targets PtdIns3P-associated with autophagosomes. A significant ABH-driven defect in autophagic pathways is also supported by co-localization of ABH with LC3 on rapamycin treatment (Fig. 3c and Supplementary Fig. 8) and further by the observation that overexpression of ABH does not affect cell viability in cells grown under normal serum replete conditions and in the absence of rapamycin (Fig. 3a). This supports that ABH may most critically target autophagy during bacterial infection.

### Non-toxigenic *V. cholerae* induces autophagy

**Figure 7:**
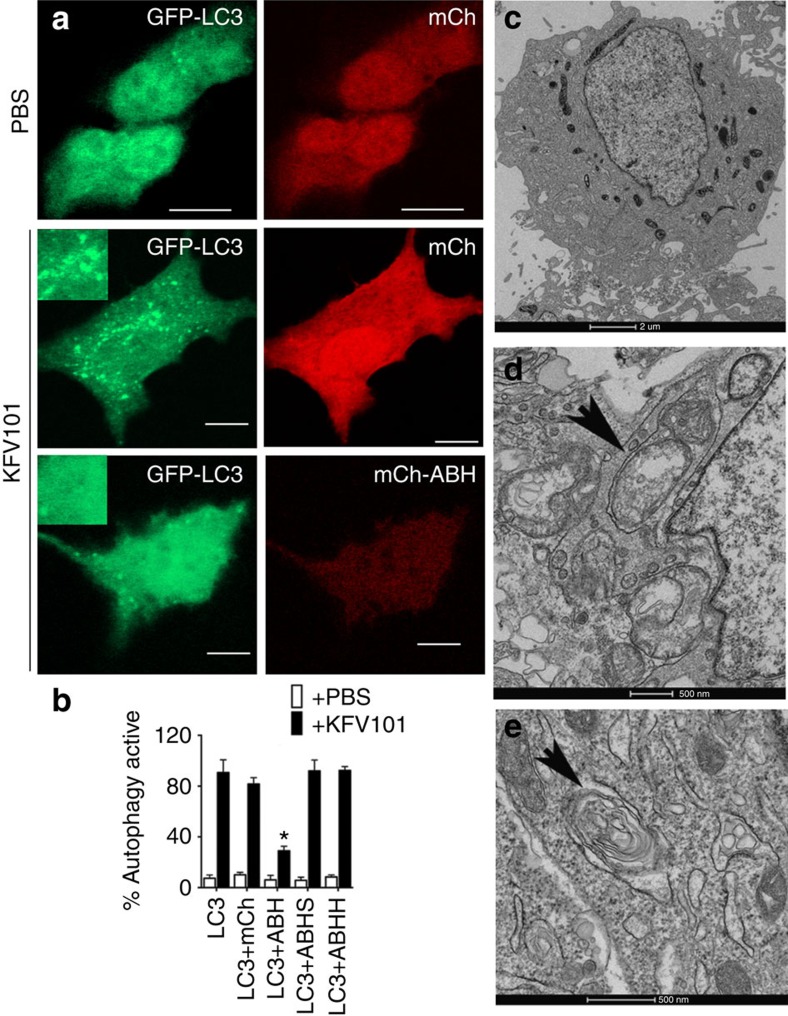
ABH inhibits *V. cholerae* induced autophagy. HeLa cells transfected for expression of LC3-GFP and stained as indicated in Fig. 3. (**a**) HeLa cells were either mock treated with PBS or co-cultured with *V. cholerae* KFV101 for 4 h at MOI of 75, fixed and imaged. Scale bar, 10 μm. Inset shows (**b**) Autophagy-positive cells were quantified and represented as mean±s.d. (*n*=60). The cells expressing mCh-ABH showed statistically significant difference from controls cells expressing GFP-LC3, mCh or mCh-ABHS (*P*<0.0001). Electron micrographs of untreated HeLa cells (**c**) and co-cultured with KFV101 (**d**,**e**). Arrows indicate autophagic profiles.

**Figure 8:**
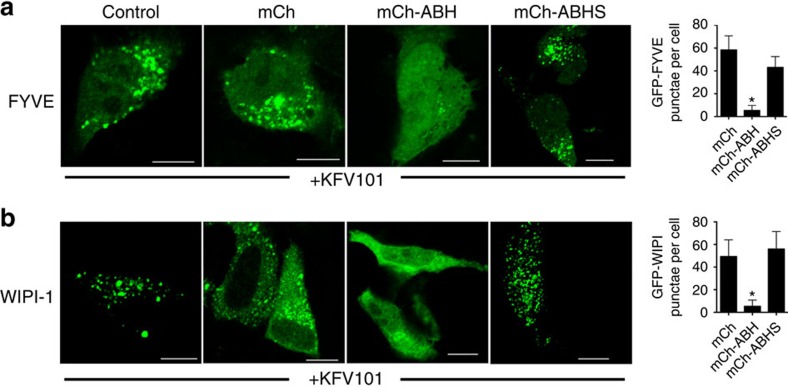
FYVE and WIPI-1 localization on bacterial infection. The cells expressing either GFP-FYVE (**a**) or WIPI-1-GFP (**b**) were either untransfected (left panels) or transfected as indicated across the top to co-express mCh, mCh-ABH or mCh-ABHS. All cells were co-cultured with *V. cholerae* KFV101 for 4 h at MOI of 75. Scale bar, 10 μm. (**e**) Cells were quantified for the number of punctae in each case and represented as mean±s.d. (*n*=45). The cells expressing mCh-ABH for both **a** and **b** showed statistically significant difference from control cells expressing only GFP-FYVE or WIPI-1-GFP or co-expressing mCh or mCh-ABHS (*P*=0.0033 and *P*=0.0055, respectively).

Bacterial control of autophagy is commonly associated with intracellular pathogens, but is also recently recognized as a critical host response to extracellular pathogens^19^,^20^,^21^. KFV101 is an El Tor O1 *V. cholerae* strain that is avirulent due to chromosomal deletions removing genes encoding cholera toxin, *V. cholerae* cytolysin (VCC), MARTX toxin and haemagglutinin/protease^4^. Compared with untreated cells, HeLa cells co-cultured with KFV101 revealed distinct evidence of undergoing autophagy. Electron micrographs showed intense vacuolization, distinct double-membrane autophagosomes engulfing cargo for degradation, and also a large autophagic vacuole with intracellular components (Fig. 7c–e). Further, cells exposed to KFV101 showed a significant increase in relocalization of GFP-LC3 to autophagosomes (Supplementary Fig. 9a) as well as punctae formation visualized by both FYVE–GFP and WIPI-1-GFP (Fig. 8). However, in cells expressing mCh-ABH, but not mCh-ABHS or mCh-ABHH, formation of autophagosomes induced by bacterial challenge was significantly inhibited reducing LC3, FYVE and WIPI-1 signal. In all cases, this was dependent on the catalytically active enzyme (Figs 7 and 8, Supplementary Fig. 9a). These data show that *V. cholerae* normally induces autophagy in cells. This induction by non-toxigenic *V. cholerae* is likely further induced by bacteria that express VCC, a toxin that also stimulates autophagy^36^. Although not the first extracellular bacteria known to induce autophagy, the control by ABH reveals that *V. cholerae* is the first recognized extracellular bacterium attempting to inhibit autophagy through use of transferred effector domains.

### ABH inhibits autophagy in context of MARTX toxin delivery

**Figure 9:**
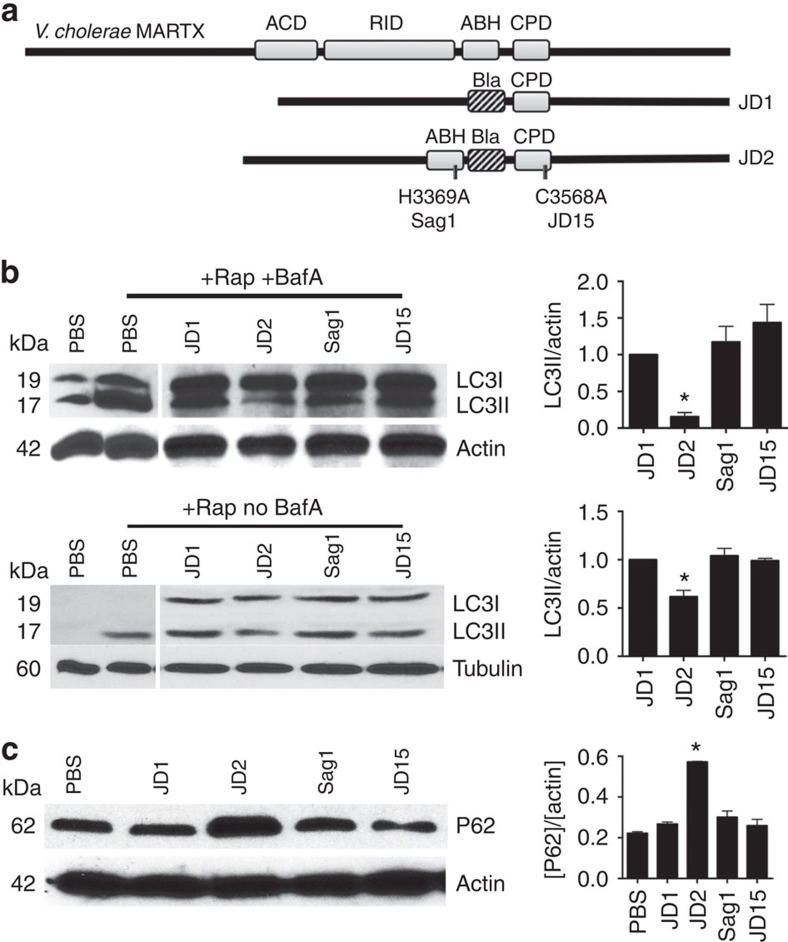
ABH inhibits host-cell autophagy in context of natural toxin delivery. (**a**) Schematic representation of MARTX_Vc_ toxin and the engineered strains harbouring modified effector domains fused with Bla. (**b**) HeLa cells were either preincubated with bafilomycin A for 1 h (+BafA, top panels) or not treated with bafilomycin A (no BafA, lower panels) followed by either mock treatment with PBS or co-culturing with the indicated bacterial strains for 4 h at MOI of 75. The challenged cells treated with 0.4 μM rapamycin (+Rap) for 12 h were lysed and probed using anti-LC3 (**b**) or anti-P62 (**c**) antibodies. Actin or tubulin was used as loading control as indicated. The intensity units were calculated by normalizing levels of LC3-II or P62 over loading control, (Intensity_Test_/Intensity_mock_)÷(actin_test_/actin_mock_) (*n*=3) using ImageJ. Values are reported as mean±s.d. (*n*=3). The LC3-II and P62 levels in the cells treated with JD2 strain (delivering wild-type ABH) showed statistically significant difference from control cells carrying Bla, ABHS-Bla or non-processing ABH-Bla (*P*<0.0001).

If ABH inhibits autophagy in response to bacterial challenge, it should do so when delivered naturally from the bacterium. Attempts to demonstrate this using *V. cholerae* producing the MARTX holotoxin would be hampered by loss of cytoskeletal structure due to actin-depolymerizing activities of ACD and RID. Thus, recently characterized *V. cholerae* strains that produce MARTX toxins that deliver only the surrogate effector β-lactamase (Bla) or Bla in combination with ABH were used (ref. 12 and Fig. 9a).

Cells undergoing autophagy lipidate LC3-I to generate LC3-II. Thus, activation of autophagy can be detected in cell lysates by its faster mobility on SDS–PAGE and western blotting using anti-LC3 antibody to detect the relative levels of LC3-I and LC3-II. HeLa cells were either left untreated or treated with vacuolar-type H^+^-ATPase inhibitor bafilomycin A1 to further enhance the assessment of autophagic flux by blocking autophagosomal maturation and clearance in lysosomes^29^.

For this assay, cells were initially co-cultured with bacteria secreting a modified toxin (Fig. 9a). Cells were then either treated with rapamycin (Fig. 9b) or serum starved (Supplementary Fig. 9b) to stimulate autophagosome formation. As expected, levels of LC3-II increased in mock treated cells stimulated with either rapamycin or serum starvation. By contrast, cells that had been treated with *V. cholerae* strain JD1 producing a toxin that translocates only the surrogate effector Bla, LC3-II was detected indicating induction of autophagy. However, in cells pretreated with JD2, a *V. cholerae* strain that translocate ABH in addition to Bla, the level of LC3-II was markedly reduced. The ability of ABH to reduce LC3-II levels depended on CPD-mediated toxin autoprocessing since LC3-II levels remained intact in cells treated with JD15, a strain that can translocate the ABH across the plasma membrane but do not deliver the domain to the cytosol due to a CPD inactivating mutation. The effect on LC3-II was also specifically due to the catalytic activity of ABH as strain Sag1 that translocates catalytically inactive ABHH did not reduce levels of LC3-II. Similar effects on LC3-II modification for all samples were obtained when the bacteria were co-incubated with cells that were not primed with bafilomycin A1 (Fig. 9b, lower panel).

Since polyubiquitin-binding protein P62/SQSTM1 is normally degraded by autophagy, there is a correlation between inhibition of autophagy and increase in P62 levels^30^. As a secondary validation of toxin-mediated inhibition of autophagy, P62 was monitored in cells treated with ABH^+^ strain JD2. These cells showed a significant increase in P62 in response to rapamycin compared with cells co-cultured with strains delivering either inactive ABH or ABH that cannot be released by autoprocessing (Fig. 9c). These results along with LC3-II accumulation results confirm that ABH can inhibit autophagy when delivered in the context of the natural toxin.

Thus, MARTX toxin translocation and delivery of the effector domain ABH by autoprocessing can functionally introduce PLA1 activity into cells to cleave PtdIns3P in preautophagosomal membranes resulting in inhibition of autophagy.

## Discussion

In the present study, we characterized the ABH effector domain of the *V. cholerae* MARTX toxin for which the molecular function was heretofore unknown. Previous bioinformatics studies had indicated that ABH belongs to the esterase/lipase hydrolase superfamily with an active site serine present in a GXSXG motif^13^. Additional structural modelling here placed this pentapeptide motif in a turn between a β-strand and α-helix, called the ‘nucleophile elbow' in other α/β hydrolases^37^,^38^. Using purified rABH, we successfully demonstrated that ABH is a highly substrate specific phospholipase A1 that strips the lipid moiety from the *sn1* position of PtdIns3P. This protein is thus the first known PtdIns3P-specific phospholipase A1 from any species.

Beyond no precedence for such an activity in cells or bacteria, the specificity of ABH for PtdIns3P was not predictable because it does not share homology with any known PtdIns3P-binding proteins. Yet, similar to other lipid binding proteins, ABH showed affinity for the anionic lipid PS, which is then greatly augmented by addition of PtdIns3P. Its 630 nM affinity for PtdIns3P is comparable to that of PtdIns3P-binding FYVE and PX domains^39^,^40^. Taking into account the fact that a high 500 mM salt concentration had to be employed to stabilize rABH for *in vitro* assays, its affinity for anionic PtdIns3P vesicles could be even higher under physiological conditions and thus have stronger affinity than cellular proteins that normally participate in initiation of autophagosome formation.

We next demonstrated that the phospholipase activity of ABH results in the reduction of intracellular PtdIns3P, likely causing the previously observed modest reduction in phagocytosis^12^ and the defects in endosomal sorting and recruitment of proteins to isolation membranes observed in this study. In addition, ABH has been linked to an activation of CDC42 (ref. 12), a small GTPase regulated by GTPase exchange factors and GTPase-activating proteins whose activation states are controlled by binding phosphoinositides other than PtdIns3P. This indicates that the ongoing specific depletion of PtdIns3P in cells by ABH may as a secondary consequence alter the equilibrium concentrations of other phosphoinositides, thereby affecting other cell biological events including the activation of CDC42 (ref. 41).

The most profound effect observed was on inhibition of autophagosome maturation both when ABH is ectopically expressed or delivered to host cells in its natural context. Although many different bacteria inhibit autophagy by various mechanisms^17^, this is the first effector that does so by uniquely binding and cleaving PtdIns3P. *Streptococcus pyogenes* SpeB protease blocks autophagy by degrading host proteins that target bacteria to autophagosomes^42^. *Legionella pneumophila* RavZ, a cysteine protease generates deconjugated Atg8 (LC3) and prevents its accumulation on autophagosomes, interfering with autophagy^43^. Thereby, both RavZ and SpeB, unlike ABH, function later in the autophagy process at the point when proteins are recruited to the autophagosome.

Recently, VipD, an effector from *L. pneumophila*, was found to have PLA1 activity against a broad array of lipids and has been demonstrated to remove endosomal PtdInsP3 (refs 44, 45). In fact, overexpression of VipD results in decreased cell viability due to the broad spectrum of lipids it hydrolyzes. However, VipD PLA1 activity *in vivo* requires stimulation by Rab5 present on early endosomes, where it has been found to affect PtdIns3P levels. However, it is notable that VipD does not displace PtdIns3P from early endosomes by being a PtdIns3P-specific enzyme, but rather its specificity is established by its ability to bind Rab5 (refs 44, 45).

Autophagic flux and PtdIns3P levels are also modulated by *Listeria monocytogenes* PI-PLC and PC-PLC^46^,^47^,^48^. These enzymes first facilitate release of *L. monocytogenes* to the cytosol by dissolving the endocytic membrane, but then prevent capture of *L. monocytogenes* into an autophagosome by reducing cellular PtdIns3P levels^46^,^47^,^48^. However, it is notable that PtdIns3P is not a substrate for PI-PLC,^47^,^49^ but instead PtdIns3P levels are indirectly reduced through the consumption of cellular levels of unphosphorylated PtdIns that would otherwise be a substrate for PI3K activation to produce PtdIns3P^48^.

Perhaps the bacterial effector closest to ABH in concept is SapM, a secreted PtdIns3P-specific phosphatase of *Mycobacterium tuberculosi*s that results in loss of PtdIns3P from phagosomes^50^. This process prevents phagosome maturation and thereby facilitates bacilli survival within a vacuole^50^. While *V. cholerae* suppression of autophagy could be achieved by a phosphatase mechanism similar to SapM, it is likely that during induction of autophagy, dephosphorylation of PtdIns3P would be easily reversed by the action of PI3K Vps34, as opposed to the irreversible removal of fatty acid from the phospholipid backbone. Thus, ABH unlike SapM has the ability to induce an irreversible inhibition of autophagy. Further, by functioning as a PLA1 instead of PLA2, ABH prevents the release of the proinflammatory molecule arachidonic acid into cells. Similarly, ABH avoids the release of signal molecule DAG, a product of *L. monocytogenes* PI-PLC activity. Thus, the unique ABH enzyme can significantly inhibit autophagy and reduce other PtdIns3P functions, without simultaneously stimulating other cellular processes.

Another major distinction of the function of *V. cholerae* ABH compared to *M. tuberculosis*, *L. pneumophila* and *L. monocytogenes* is that inhibition of endosome maturation by these bacteria is essential to provide a vacuole for their intracellular survival^17^,^18^. However, only rare non-O1 *V. cholerae* isolates harbouring a Type III secretion system are reported to be invasive^51^. Thus the need to control autophagy by the more common *V. cholerae* El Tor O1 used in this study is not likely to be a consequence of intracellular invasion. In fact, purified recombinant VCC encoded by the *V. cholerae* gene *hlyA* can induce autophagy^36^. Since MARTX and VCC work in concert^4^, ABH might contribute to reduction of VCC-induced autophagy when both toxins are present.

Yet, we found that a strain that does not produce VCC or any other secreted accessory toxins can still induce autophagy suggesting *V. cholerae* stimulates autophagy by other mechanisms. Recently, extracellular pathogen *Pseudomonas aeruginosa* has been shown to induce autophagy both through iron chelation and through activation of ERK in *Caenorhabditis elegans* and through lipopolysaccharide stimulation of TLR4 (refs 19, 52). Peptidoglycan present in outer membrane vesicles has been shown to induce autophagy by stimulating Nod1 and RIP1 in early endosomes^53^. The subsequent autophagy-dependent activation of innate signalling results in production of proinflammatory chemokines and cytokines and thereby improves pathogen clearance^19^,^20^,^21^. *V. cholerae* is herein also recognized as an extracellular pathogen that stimulates autophagy, but also is now the first extracellular bacterium found to produce a toxin that blocks this innate response via the unique mechanism of MARTX-dependent destruction of PtdIns3P by the PLA1 activity of ABH.

## Methods

### Plasmid, bacterial strains and cell culture

HeLa cells (purchased from ATCC) were grown at 37 °C/5% CO_2_ in DMEM (Invitrogen) with 10% FBS (Invitrogen), 50 μg ml^−1^ penicillin, 50 μg ml^−1^ streptomycin. *Escherichia coli* (Invitrogen)-TOP10 and BL21(λDE3) were cultured at 37 °C in Luria Broth (LB) or agar plates containing 50 μg ml^−1^ kanamycin or 100 μg ml^−1^ ampicillin. GFP-LC3 (#24920) and pEF1α-mCherry-N1 (#631969) plasmids were procured from Addgene and Clontech, respectively. APP-GFP plasmid was constructed as described^31^. The GFP-2 × -FYVE^HRS^ plasmid was a gift from Harald Stenmark (University of Oslo, Norway). GFP-WIPI-1 was a gift from Sharon Tooze (Cancer Research UK).

### Characterization of recombinant ABH and its variants

Wild-type ABH and its catalytic variants were cloned, expressed and purified from *E. coli*. *V. cholerae* N16961 *rtxA* DNA corresponding to the ABH domain was amplified from genomic DNA and the 810-bp product cloned in pET-15b (Novagen) using BamH1 and XhoI enzymes to generate plasmid pME14. Site-directed mutagenesis according to Agilent QuickChange protocol was performed on pME14 to replace alanine codons for Ser-3259 (TCA-GCT), His-3369 (CAC-GCT) and Asp-3338 (GAT-GCT). All plasmid inserts were sequenced to confirm accuracy of PCR and mutagenesis. *E. coli* BL21(λDE3) transformed with pME14 or plasmids with point mutations were grown in LB with 100 μg ml^−1^ ampicillin to *A*_600_=0.6–0.8 and protein expression was induced by 4 h growth in 1 mM isopropyl-β-D-thiogalactopyranoside. 6 × His-tagged rABH proteins were purified using Talon affinity cobalt resin (Clontech), the protein was dialysed in 20 mM Tris-HCl, pH 8.0, 500 mM NaCl, and stored at −80 °C till further use. Rabbit polyclonal antibody against rABH was generated by Lampire Biological Laboratories (Pipersville, PA) using a 50-day express-line protocol.

### Circular dichroism and thermal shift assay

Secondary and tertiary structural differences between wild type and mutants, if any, were assessed using Circular dichroism and thermal shift assays. Far-ultraviolet CD spectra (200–250 nm) of 50 μg ml^−1^ wild type and mutant ABH in 50 mM Tris-HCl, pH 8.0 and 500 mM NaCl were acquired at 25 °C in a 0.1-cm cuvette at 50 nm per min speed using Jasco J-815 Spectropolarimeter. Five individual spectra were averaged for each sample. The CD signals were de-convoluted by CONTIN software^54^.

For determination of *T*_m_, 20 μl reactions of wild type and mutant proteins (1 mg ml^−1^) were incubated with the 1 × Life Technologies SYPRO orange dye and analysed as described^55^.

### Esterase assay

One millimolar pNP-acetate (C2), pNP-butyrate (C4), pNP-caprylate (C8), pNP-palmitate (C16) obtained from Sigma were incubated with 0.8 μM rABH proteins at 37 °C for 30 min in 100 μl TT buffer (50 mM Tris-HCl, 0.5% Triton X-100, pH 8.0). pNP release was monitored using a Beckman DU540 spectrophotometer at *A*_405_ nm. Specific activity (pmoles pNP released per min) was calculated using the molar extinction coefficient of pNP as 5,150 M^−1^ cm^−1^.

### Lipase assay

DAG, PC, phosphatidylethanolamine, phosphatidylglycerol, PtdIns, PtdIns3P, phosphatidyl inositol 3,4 bis-phosphate (PtdIns3,4P(2)), phosphatidyl inositol triphosphate (PtdIns3,4,5P(3)) were obtained from Avanti Polar Lipids (Alabama) and D-L-α-palmitin (1-MG) from Sigma. Hundred micrograms of lipid was incubated with 8 μM rABH proteins in TT buffer at 37 °C for time indicated. FFA was measured using the Wako Diagnostics HR Series NEFA-HR(2) colorimetric assay according to manufacturer's instructions and the specific activity was calculated using the NEFA standard solution.

### Membrane lipid overlay assay

Echelon Biosciences PIP Strips were incubated with 80 nM rABH proteins in PBS containing 0.25% Tween (PBST) and 2% powdered skimmed milk (SM-PBST) overnight at 4 °C followed by three washes in 1 × -PBST buffer. rABH was detected using anti-6xHis monoclonal antibody (1:5,000, Sigma, #H1029) followed by anti-mouse IgG-horseradish peroxidase (HRP) conjugated antibody (1:10,000, Jackson Laboratories, #111-035-003). The blot was developed using chemiluminescence substrate solution (Pierce) and X-ray film.

### Surface plasmon resonance (SPR) analysis

Lipids dissolved in chloroform were dried under nitrogen and resuspended in 500 μl 20 mM Tris-HCl, 500 mM NaCl, pH 7.4. Mixture was vortexed for 25 min at room temperature and sonicated for 1 min in a Branson 1,200 sonifier. Large unilamellar vesicles with 100-nm diameter were prepared with a microextruder (Avanti Polar Lipids) using a 100-nm polycarbonate filter. SPR measurements were performed at 23 °C using lipid-coated L1 chip in the BIACORE X system^56^. Briefly, after washing the sensor chip with buffer (20 mM Tris-HCl, pH 7.4, 0.5 M NaCl), PC/PS/PtdIns3P (or PtdIns4P) (77:20:3) and 1,2-dioleoyl-*sn*-glycero-3-ethylphosphocholine (EPC; Avanti Polar Lipids) vesicles were injected at 5 ml min^−1^ to the active and the control surface, respectively. Assuming a Langmuir-type binding between the protein (P) and protein binding sites (M) (that is, P+M↔PM), *R*_eq_ values were plotted versus *P*_*0*_, and the *K*_d_ value was determined by a nonlinear least-squares analysis of the binding isotherm using an equation, *R*_eq_=*R*_max_/(1+*K*_d_/*P*_*0*_) (ref. 40). Each data set was repeated three times for statistical significance.

### Mass spectrometry

1-heptadecanoyl-2-(5Z,8Z,11Z,14Z-eicosatetraenoyl)-*sn*-glycero-3-phospho-(1′-myo-inositol-3′- phosphate; Avanti Polar Lipids) was treated with rABH proteins as described above. The lipid samples were analysed on an Agilent 6,490 Triple Quadrupole LC/MS instrument. Samples were injected via autosampler into a chloroform/methanol (1:1(vol/vol), 20 mM piperidine) solvent (flow rate 0.25 ml min^−1^). The nozzle voltage, capillary voltage and source gas parameters were set at −1.5 kV, −2 kV and 200 °C, respectively for single stage ion profiling. Substrate identities were identified by MS/MS experiments using similar parameters except for higher collision energies to enable substrate fragmentation, 35–40 eV for lyso-PtdIns3P and 43 eV for PtdIns3P.

### Confocal microscopy

HeLa cells seeded overnight at 60–70% confluency onto 35 mm^2^ coverslips were transfected with DNA:Roche FUGENE mix (1:3) according to manufacturer's instructions. After 18–20 h coverslips were fixed and stained with 0.35 μM 4,6-diamino-2-phenylindole dihydrochloride (Invitrogen) as described^9^. Cells were imaged at × 1,000 using either a Zeiss LSM510 META-UV or a Zeiss LSM700 confocal microscope. Co-localization was quantified using Nikon C2 software or Image J Software (NIH). After 18–20 h of transfection, the medium was exchanged with either phenol free DMEM containing 10% FBS and 1 × penicillin–streptomycin with 0.4 mM rapamycin dissolved in DMSO or 5 mM 3-methyladenine for 1 hr or HBSS for 12 h. Quantification of GFP-LC3 punctae in each case as indicated was calculated using the following formula:% Autophagy Active=(number of cells with >7 punctae/total number of cells) × 100.

The threshold of 7 was based on an observed 3–5 punctae in cells treated with DMSO alone. The number of GFP-FYVE and WIPI-1-GFP punctae were quantified in each case and the average size of the punctae in GFP-FYVE was obtained by dividing the surface area of the FYVE-positive compartment (in pixel^2^) by the number of FYVE punctae using Image J software. APP-GFP peripheral versus internal localization was quantified from 20–24 cells in two different experiments with an average quantification of seven giant endosomes per cell.

### Western blotting

Cells grown in 12-well culture plates were lysed in 200 μl of 2 × SDS–PAGE buffer, separated by SDS–PAGE, and transferred to nitrocellulose. For loading controls, blots were either cut and halves of same blot developed with different antibodies or blots were stripped and reprobed as indicated in legends and supplement. Immunoblotting was conducted using monoclonal anti-GFP antibody coupled to HRP (1:1,000, Miltenyi Biotec Inc., CA, #130-091-833) or using primary antibodies as follows: custom raised rabbit polyclonal anti-ABH (1:1,000, Lampire Biological Laboratories, Pipersville, PA), polyclonal rabbit anti-LC3 (1:10,000, Novus Biologicals, #NB100-2,220), polyclonal guinea pig anti-P62 (1:5,000, Progen Biotechnik, #GP62-C), polyclonal rabbit anti-actin (1:5,000, Sigma, #A4700) or mouse monoclonal anti-tubulin (1:5,000, Sigma, #T5168). Secondary HRP-conjugated goat antibodies against rabbit (1:5,00, Jackson Laboratories, #111-035-003), mouse (1:5,000, Sigma, #A2304) or guinea pig (1:5,000, Sigma, #A7289) were used to develop blots using chemiluminescence substrate solution (Pierce) and X-ray film.

### Bacterial co-culture experiments

*V. cholerae* strains KFV92, JD1, JD2, Sag1, JD15 (ref. 12) were grown in LB containing 100 μg ml^−1^ streptomycin with shaking at 37 °C. HeLa cells were either untreated or treated with 160 nM bafilomycin A (Sigma) and simultaneously co-cultured with the bacterial strains for 4 h followed by addition of 100 μg ml^−1^ of gentamicin for 1 h at 37 °C. HeLa cells were treated either with 0.4 μM rapamycin in phenol red-free DMEM or HBSS for 12 h at 37 °C/5% CO_2_ (to amplify autophagy induction). The cells were lysed in 250 μl of 2 × SDS–PAGE buffer and the levels of LC3 or P62 were quantified by western blotting as described above.

### Transmission Electron Microscopy

HeLa cells were co-cultured with *V. cholerae* at MOI 75 for 4 h and then fixed in 0.1 M sodium cacodylate buffer pH 7.3 containing 2% paraformaldehyde and 2.5% glutaraldehyde. Post fixation of the samples was done with unbuffered 1% osmium tetroxide, en-bloc stained with 2% uranyl acetate, transitioned with propylene oxide and embedded in resin mixture of Embed 812 kit. Samples were sectioned on a Leica Ultracut UC6 ultramicrotome. Sections (70 nm) were collected on 200 mesh copper grids; stained with uranyl acetate and reynolds lead citrate and were visualized using FEI Tecnai Spirit G2 transmission electron microscopy.

### Statistical analysis

All experiments were performed at least in triplicates. Quantitative results are reported as the mean±s.d. Statistical significance between samples was determined by one-way ANOVA followed by multiple comparison's using the GraphPad Prism 6.0 software.

## Additional information

**How to cite this article:** Agarwal, S. *et al*. Autophagy and endosomal trafficking inhibition by *Vibrio cholera* MARTX toxin phosphatidylinositol-3-phosphate-specific phospholipase A1 activity. *Nat. Commun.* 6:8745 doi: 10.1038/ncomms9745 (2015).

## Supplementary Material

Supplementary Information Supplementary figure 1-14
