# Erratum: Autophagy and endosomal trafficking inhibition by *Vibrio cholerae* MARTX toxin phosphatidylinositol-3-phosphate-specific phospholipase A1 activity

**DOI:** 10.1038/ncomms10135

**Published:** 2015-11-27

**Authors:** Shivani Agarwal, Hyunjin Kim, Robin B. Chan, Shivangi Agarwal, Rebecca Williamson, Wonhwa Cho, Gilbert Di Paolo, Karla J. F. Satchell

Nature Communications 6: Article number: 8745 10.1038/ncomms9745 (2015); Published: 10262015; Updated: 11272015

The original version of this Article contained an error in the spelling of the author Gilbert Di Paolo, which was incorrectly given as Gilbert D. Paolo. This has now been corrected in both the PDF and HTML versions of the Article.

