# Supplementary material for: Autophagy and endosomal trafficking inhibition by Vibrio cholerae MARTX toxin phosphatidylinositol-3-phosphate-specific phospholipase A1 activity
Source: Nat Commun. 2015 Oct 26;6:8745. doi: 10.1038/ncomms9745 (PMC4640098; doi:10.1038/ncomms9745)
Supplement: Supplementary Information — Supplementary figure 1-14 [file ncomms9745-s1.pdf]

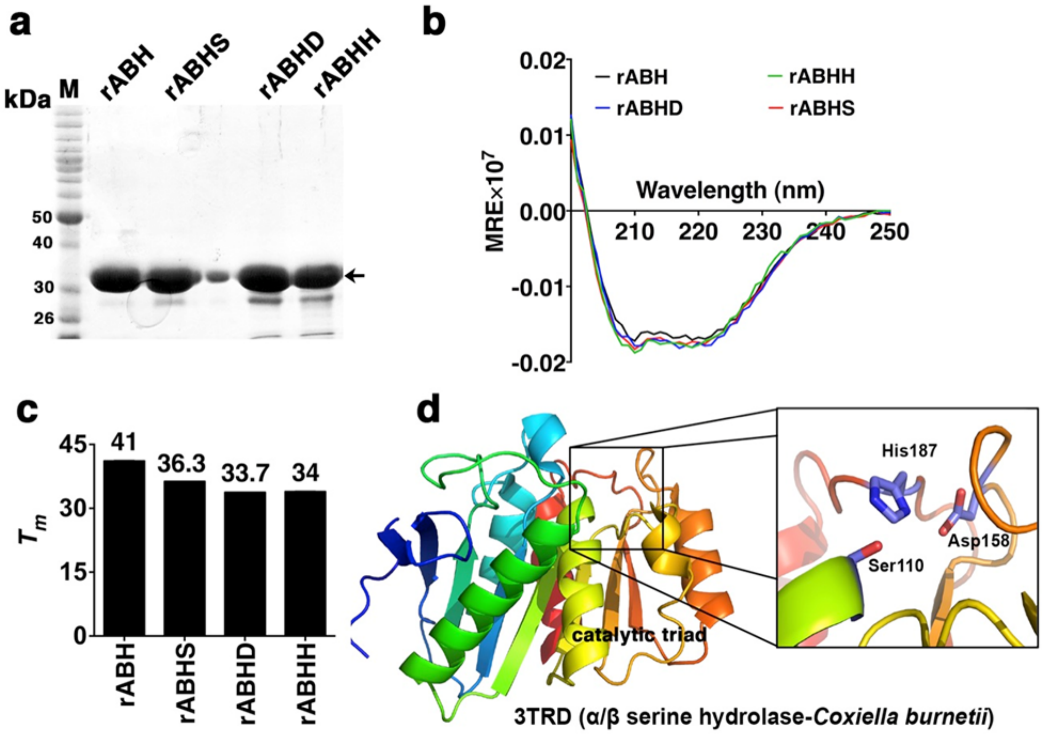

Supplementary Figure 1 │ Structural modeling and purification of *V. cholerae* ABH. (a) The migration of the purified rABH and catalytically inactive variants rABHS, rABHD, and rABHH are shown on 12% SDS-PAGE with molecular weight marker (M). (b) Average CD spectra of rABH proteins. (c) The T_m of the wild type and mutant ABH proteins generated by the thermal melting analysis. (d) The amino acid sequence of ABH was aligned to structural database using HHPRED (http://www.ncbi.nlm.nih.gov/pubmed/19626712) revealing the crystal structure of serine hydrolase of *Coxiella burnetti* (PDB: 3TRD) as 30% identical to ABH. Highlighted are the catalytic serine, aspartate and histidine residues forming a catalytic cleft. An *in silico* model of ABH was build on this model (not shown) to identify putative catalytic residues.

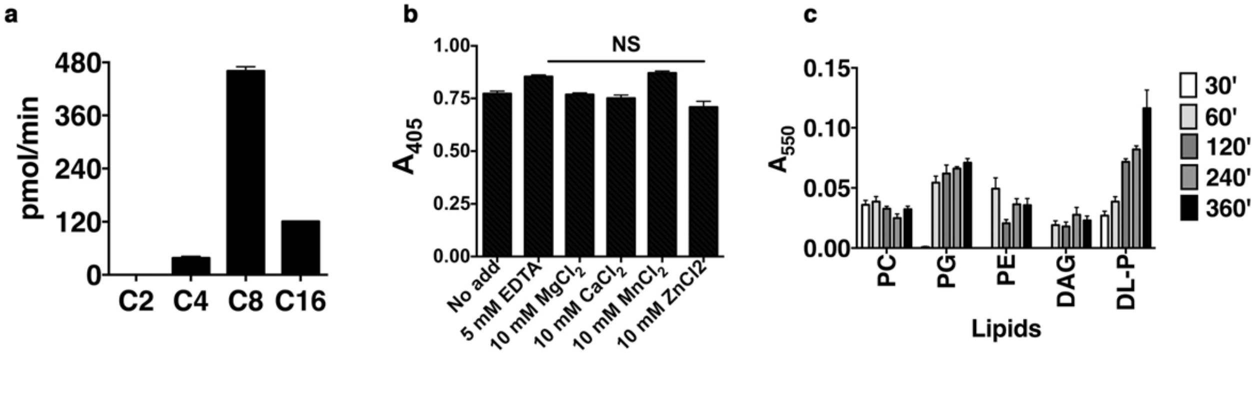

Supplementary Figure 2 │ ABH is a specific esterase/lipase.

(a) Ester hydrolysis by rABH with pNP-C2/C4/C8/C16 for 30 min. The values represent the mean ± SD (*n*=3) tested independently. (b) Ester hydrolysis by rABH with pNP-C8 for 30 min in presence of EDTA or cations as indicated. (c) The release of FFAs from the phospholipid substrates at the indicated time points in the presence of rABH. All values represent the mean±SD derived from three independent *in vitro* reactions.

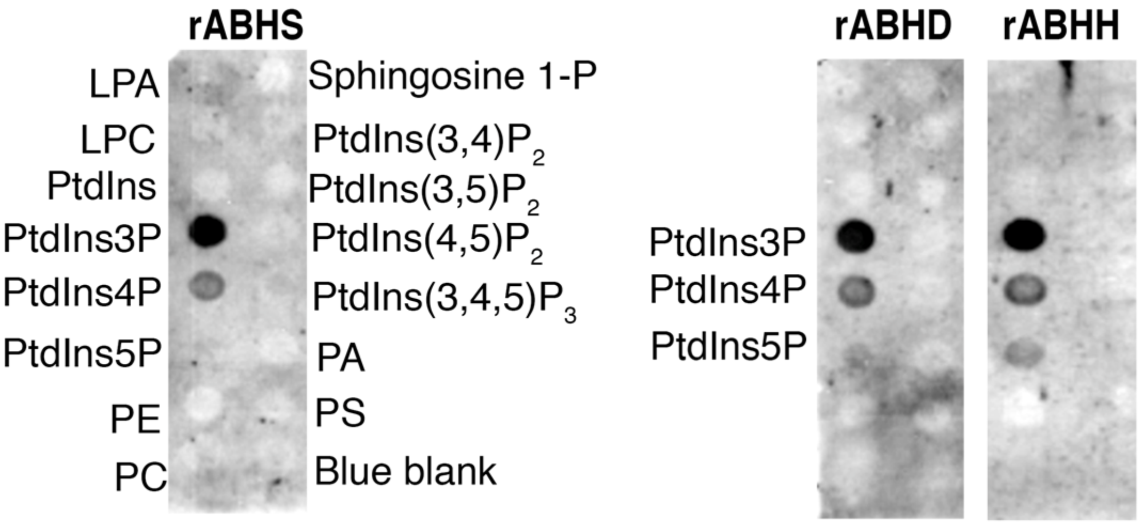

Supplementary Figure 3 | ABH interaction with PtdIns3P

PIP strip pre-coated with indicated lipids incubated with catalytic variants of rABH and probed with anti-His monoclonal antibody.

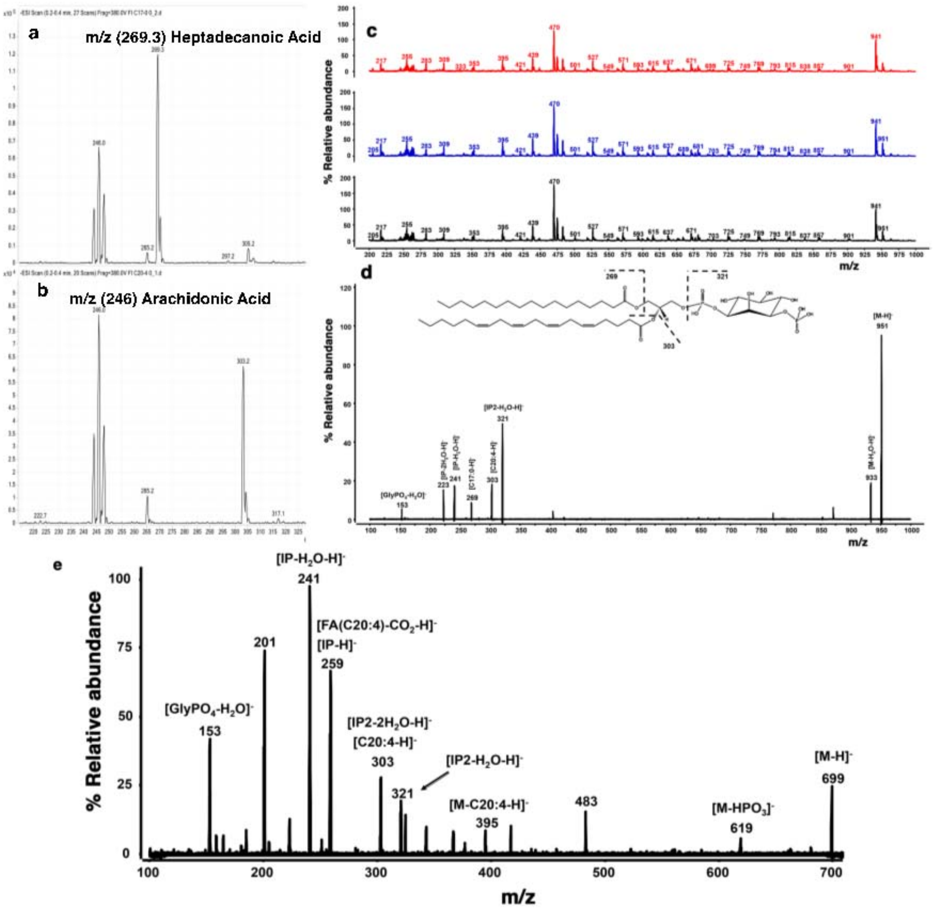

Supplementary Figure 4 │ ABH is PtdIns3P specific PLA1.

Mass spectrometric profiles of commercially available heptadecanoic acid (a) and arachidonic acid (b) used as reference for the peaks obtained with the experimental sample containing ABH. (c) Representative single stage ion profile of substrate (black) with rABH (red) and rABHS (blue). Spectra were normalized to the PtdIns3P 36:2 (941 m/z) peak. (d) Determination of molecular identity of substrate PtdIns3P 37:4 via tandem MS/MS. (e) Determination of the molecular identity of peak 699 m/z via tandem MS/MS. The mass spectra obtained with different collision energies were merged to form the composite mass spectrum. M, lysoPtdIns3P20:4; IP, inositol monophosphate; IP2, inositol bisphosphate; C20:4, arachidonate; $GlyPO_4$, cyclic glycerophosphate. Diagnostic daughter ions are labeled in the spectrum in both panels d and e.

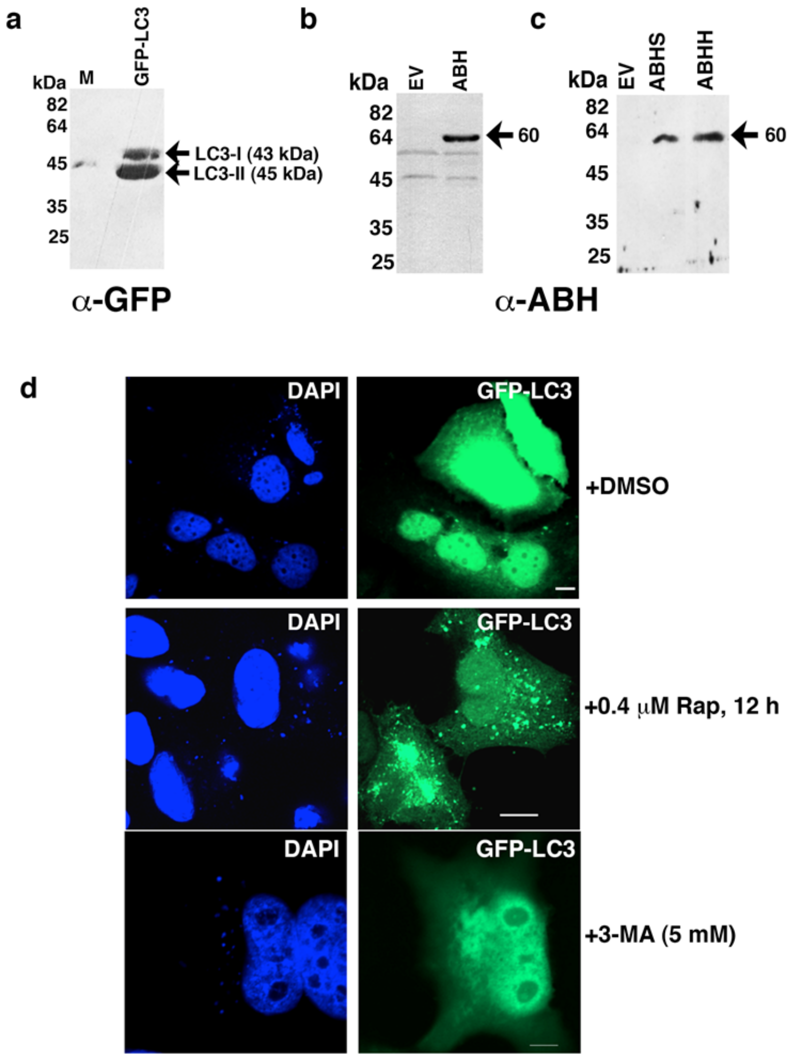

Supplementary Figure 5 │ ABH inhibits rapamycin-induced autophagy.

(a) Western blot detection of GFP-LC3 in the transfected HeLa cells probed with anti-GFP antibody showing mobility of both GFP-LC3I and GFP-LC3-II on a 12% SDS-PAGE with molecular weight standard (M). (b, c) Western blot detection of mCh-ABH expression in HeLa cells transfected as indicated probed with custom-raised antibody against rABH. EV indicates empty vector. (d) Representative fluorescence images of HeLa cells transfected with GFP-LC3 treated with rapamycin, 3-methyladenine or vehicle control DMSO. The cell nuclei were stained with DAPI. Bar=5 μm.

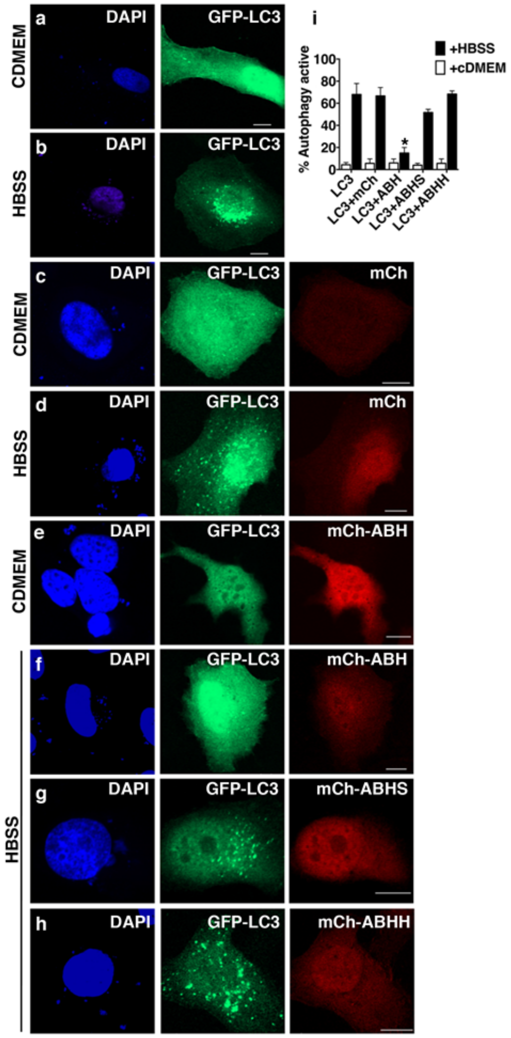

Supplementary Figure 6 │ ABH inhibits autophagy induced by serum starvation.

Confocal microscopy of HeLa cells transfected to express GFP-LC3 alone (green) or co-transfected to express mCh, mCh-ABH, mCh-ABHS, or mCh-ABHH as indicated (red). The cell nuclei were stained with DAPI (blue). The cells were incubated with either cDMEM as control (a, c, e) or HBSS as nutrient deprivation (b, d, f-h) for 12 h. (i) Autophagy positive cells were quantified as described in methods. The values represent mean±SD of three independent samples. Bar=10 µm. Asterisk indicates statistically significant difference from all other samples ($p<0.001$).

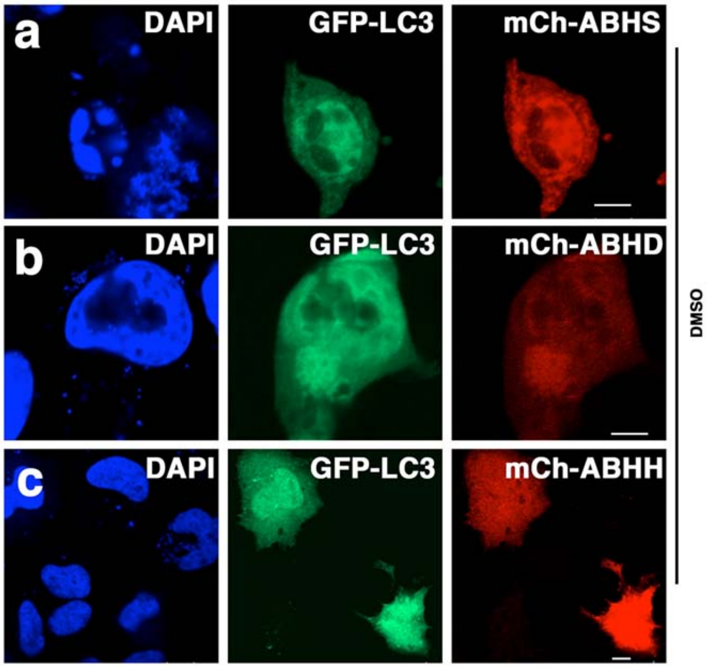

Supplementary Figure 7 │ Catalytic variants of ABH do not inhibit autophagy. Confocal microscopy of HeLa cells co-transfected to express GFP-LC3 and mCh-ABH catalytically inactive mutants in the presence of DMSO. The cell nuclei were stained with DAPI. Bar=10μm.

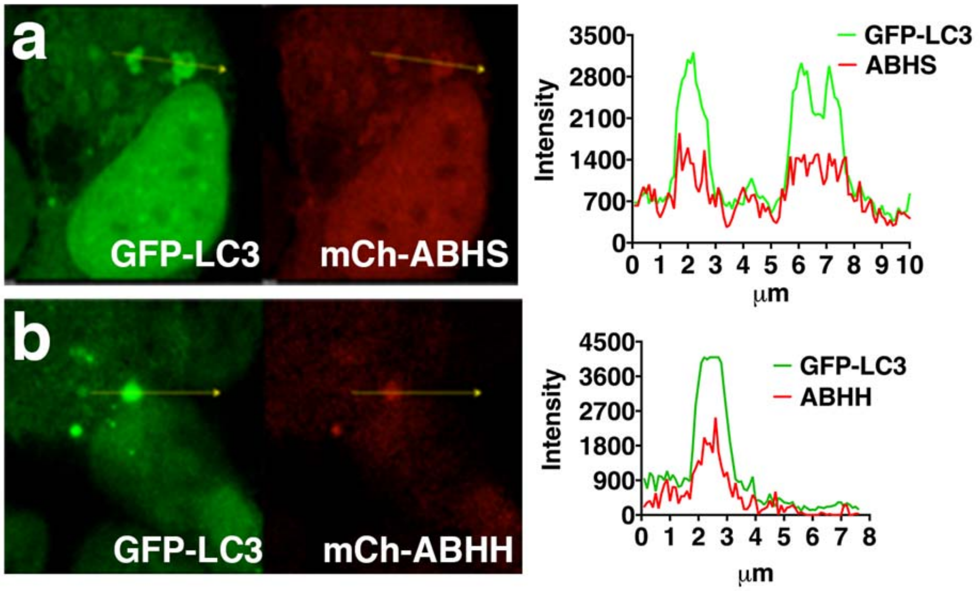

Supplementary Figure 8 │ Catalytic mutants of ABH localize to autophagosomes. Co-localization of GFP-LC3 (green) with mCh-ABHS (a) and mCh-ABHH (b) in HeLa cells treated with rapamycin. The graphs show the intensity (*y*-axis) of mCh-ABHS/H (red) and GFP-LC3 (green) against the μm distance (*x*-axis) along the yellow arrow in the image. Analysis shown is representative of at least 3 spots analyzed.

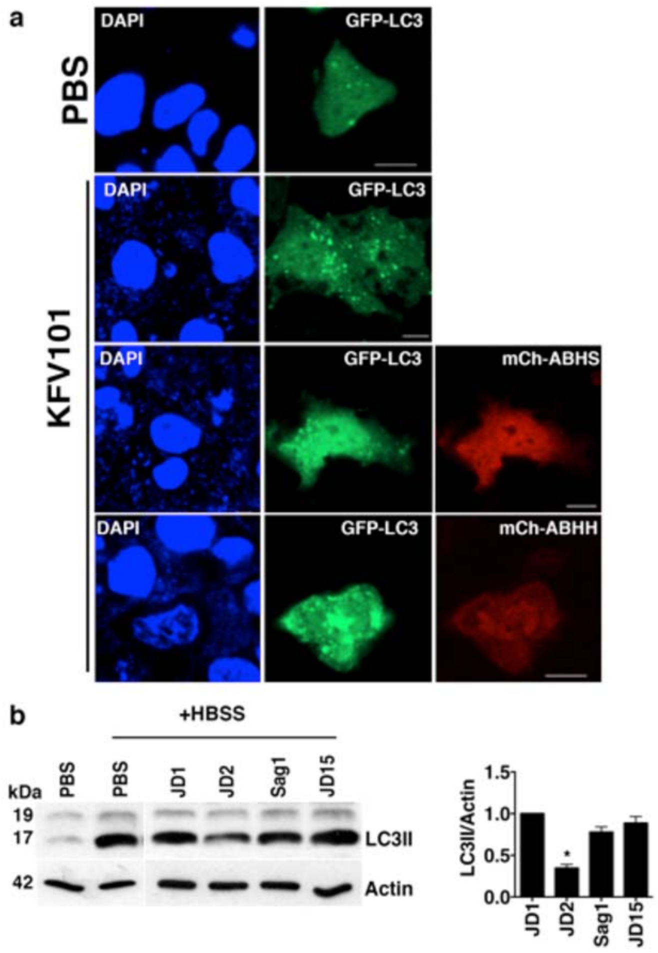

Supplementary Figure 9 │ ABH inhibits autophagy induced by bacterial challenge. (a) Confocal microscopy of HeLa cells transfected and stained as indicated. The cells were co-cultured with *V. cholerae* KFV101 for 4 h at MOI of 75. Note that DAPI also stains DNA of bacteria (comma-shape). Bar=10 µm. (b) HeLa cell were pre-incubated with bafilomycin A for 1 h followed by either mock treatment with PBS or co-culturing with the indicated bacterial strain for 4 h at MOI of 75. The challenged cells were then treated with HBSS for 12 h. Collected lysates were then analyzed by western blotting using anti-LC3 antibody and anti-actin antibody detection of a stripped blot was used as a loading control. Graph represents the normalized intensity units using Image J software. The intensity units were calculated by plotting the normalized levels of LC3-II calculated as $(\text{Intensity}_{\text{Test}}/\text{Intensity}_{\text{mock}}) \div (\text{actin}_{\text{test}}/\text{actin}_{\text{mock}})$. Values are reported as mean ± SD (*n*=3). Asterisk indicates statistically significant difference compared to all other samples ($p<0.0001$).

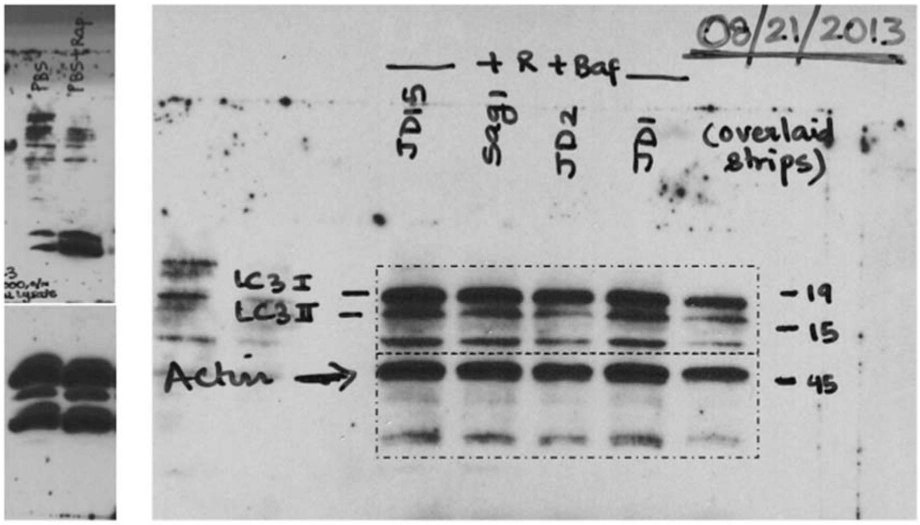

Supplementary Figure 10 │ Full length Western blot of Figure 9b.

Panel on top left shows the control samples probed with anti-LC3 and the blot below that are control samples probed with anti-actin antibody. The arrow indicates the protein. The boxed areas on the central blot represents the two thin strips with experimental samples (as labeled) probed with LC3 and actin antibodies. The migration of marker is also shown. The samples were loaded and the blot was cut into thin strips separating the regions of low molecular weight (for LC3 staining) and high molecular weight (for actin). The blots were developed on one X-ray film. The control samples were separated from the experimental samples due to the difference in the amount of protein expressed.

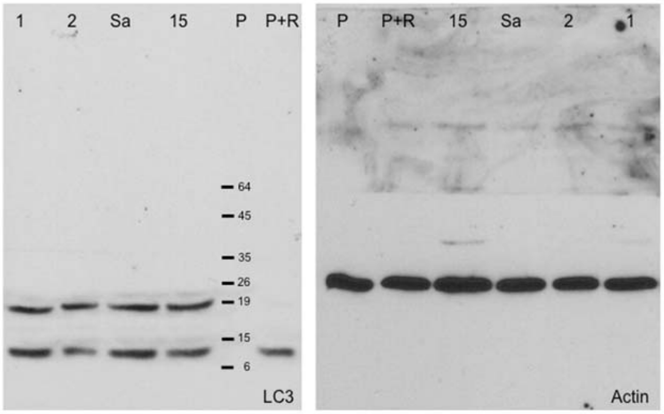

Supplementary Figure 11│ Full size Western blot of Figure 9b.

Panel on top left shows the control and experimental samples (1=JD1; 2=JD2; Sa=Sag1; 15=JD15; P+R=PBS with Rapamycin; P=PBS alone) probed with anti-LC3 and the blot on right are the same control and experimental samples probed with anti-actin antibody.

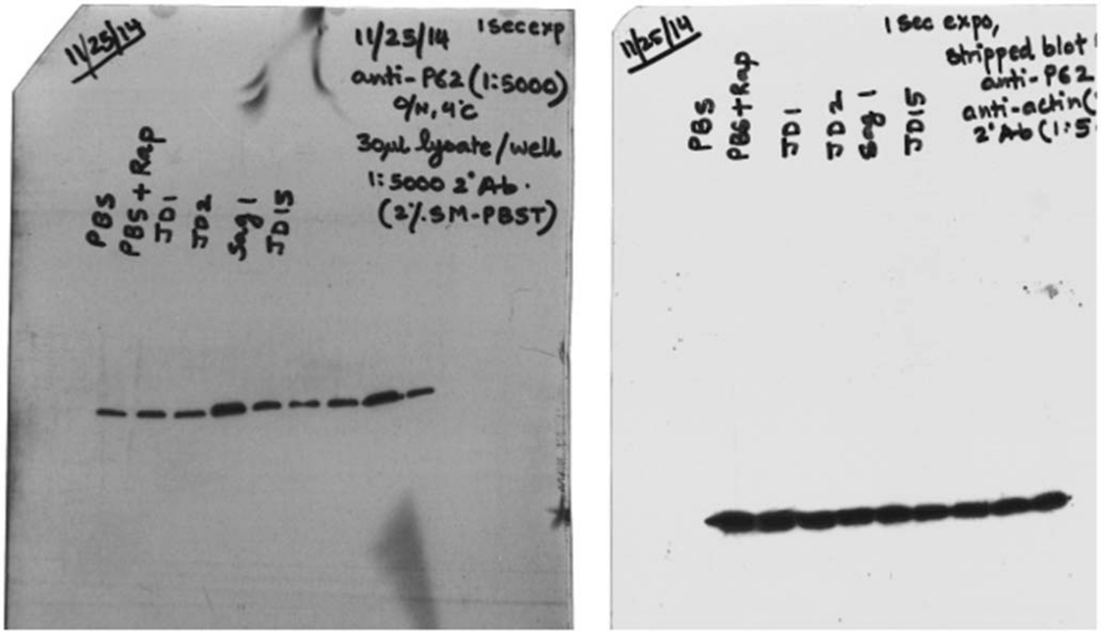

Supplementary Figure 12 | Full size Western blot of Figure 9c.

Panel on top left shows the control and experimental samples probed with anti-P62 antibody and the blot on right are control and experimental samples probed with anti-actin antibody. The P62 blot was stripped and developed for the actin loading control.

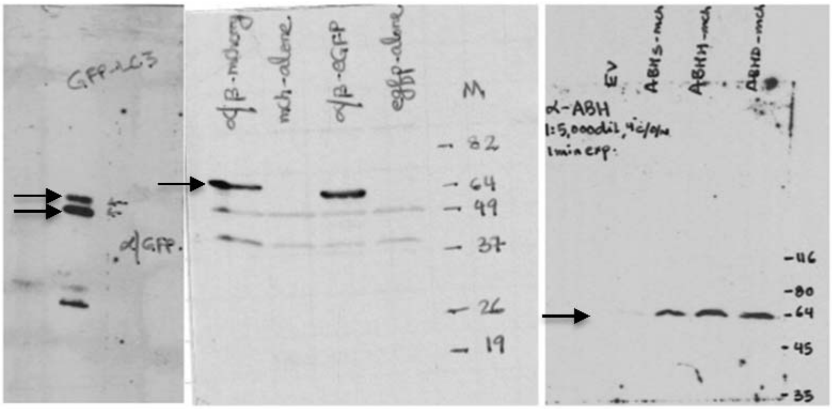

Supplementary Figure 13│ Full size Western blot of Supplementary Figure S5.

Panel on top left shows the cell lysates from cells expressing GFP-LC3 probed with anti-GFP antibody, middle panel is cells expressing mCh alone or mCh-ABH and probed with anti-ABH antibody, right panel shows lysates expressing ABHS, ABHD and ABHH probed with anti-ABH antibody. EV indicates the cells expressing empty vector, mCh. Arrows point to the proteins of interest.

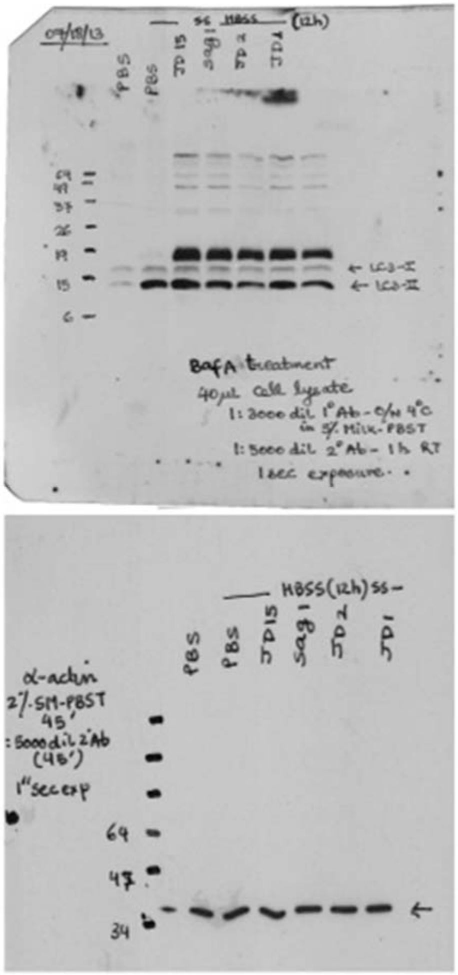

Supplementary Figure 14│ Full length Western blot of Supplementary Figure 9b.

Panel on top left shows the control and experimental samples (as indicated) probed with anti-LC3 and the blot below are the same control and experimental samples probed with anti-actin antibody. Arrows top gel show the LC3 isoforms.
